# Gram-Positive Bacterial Extracellular Vesicles and Their Impact on Health and Disease

**DOI:** 10.3389/fmicb.2018.01502

**Published:** 2018-07-09

**Authors:** Yue Liu, Kyra A. Y. Defourny, Eddy J. Smid, Tjakko Abee

**Affiliations:** Food Microbiology, Wageningen University & Research, Wageningen, Netherlands

**Keywords:** Actinobacteria, Firmicutes, membrane vesicles, pathogenicity, EV vaccination, phage therapy, probiotics

## Abstract

During recent years it has become increasingly clear that the release of extracellular vesicles (EVs) is a feature inherent to all cellular life forms. These lipid bilayer-enclosed particles are secreted by members of all domains of life: Eukarya, Bacteria and Archaea, being similar in size, general composition, and potency as a functional entity. Noticeably, the recent discovery of EVs derived from bacteria belonging to the Gram-positive phyla Actinobacteria and Firmicutes has added a new layer of complexity to our understanding of bacterial physiology, host interactions, and pathogenesis. Being nano-sized structures, Gram-positive EVs carry a large diversity of cargo compounds, including nucleic acids, viral particles, enzymes, and effector proteins. The diversity in cargo molecules may point to roles of EVs in bacterial competition, survival, material exchange, host immune evasion and modulation, as well as infection and invasion. Consequently, the impact of Gram-positive EVs on health and disease are being revealed gradually. These findings have opened up new leads for the development of medical advances, including strategies for vaccination and anti-bacterial treatment. The rapidly advancing research into Gram-positive EVs is currently in a crucial phase, therefore this review aims to give an overview of the groundwork that has been laid at present and to discuss implications and future challenges of this new research field.

## Gram-Positive Bacterial EVs: an Upcoming Research Area

Although discovered 30 years later than their Gram-negative counterparts, Gram-positive bacterial extracellular vesicles (EVs) have been drawing more attention in recent years (Brown et al., 2015; Kim et al., 2015). Budding events of spherical particles and their release into the surrounding environment of the cells have been observed for a wide range of bacterial species belonging to the Gram-positive phyla Firmicutes and Actinobacteria (**Table 1**). These particles could be isolated using common EV isolation strategies and reflected lipid bilayer-enclosed structures that are morphologically similar to Gram-negative or eukaryotic EVs (Lee et al., 2009; Lee J.H. et al., 2013; Lee J. et al., 2013; Brown et al., 2014; Olaya-Abril et al., 2014; Haas and Grenier, 2015; Schrempf and Merling, 2015; Kim J.H. et al., 2016; Resch et al., 2016; Jeon et al., 2017). Consistent with other classical EVs, Gram-positive EVs were within a nano-scale size range of about 10–400 nm.

**Table 1:** Gram-positive organisms for which EV release has been demonstrated.

Phyla	Species	Evidence
Firmicutes	*Staphylococcus aureus*	TEM of budding events and EV isolates, protein characterization (SDS-PAGE, MS), fluorescence microscopy (lipid staining) (Lee et al., 2009; Gurung et al., 2011)
	*Streptococcus pneumoniae*	TEM of EV isolates, SEM of budding events, protein characterization (SDS-PAGE, MS), lipid characterization (Olaya-Abril et al., 2014)
	*Streptococcus mutans*	TEM of EV isolates, protein characterization (SDS-PAGE) (Liao et al., 2014)
	*Streptococcus suis*	TEM of cell culture and EV isolates, protein characterization (MS) (Haas and Grenier, 2015)
	*Streptococcus pyogenes/Group A streptococci*	TEM, SEM, and AFM of budding events and EV isolates, protein characterization (SDS-PAGE, MS), lipid characterization (Resch et al., 2016)
	*Streptococcus agalactiae/Group B streptococci*	TEM, SEM, and AFM of EV isolates or budding events, protein characterization (SDS-PAGE, MS), lipid characterization (Surve et al., 2016)
	*Listeria monocytogenes*	TEM of EV isolates, protein characterization (MS) (Lee J.H. et al., 2013)
	*Propionibacterium acnes*	TEM of EV isolates, protein characterization (SDS-PAGE, MS) (Jeon et al., 2017)
	*Bacillus anthracis*	TEM of EV isolates, flow cytometry (Rivera et al., 2010)
	*Bacillus subtilis*	TEM and SEM of budding events and EV isolates, protein characterization (SDS-PAGE, MS) (Brown et al., 2014; Kim Y. et al., 2016)
	*Clostridium perfringens*	TEM of EV isolates, protein characterization (SDS-PAGE, MS) (Jiang et al., 2014)
	*Lactobacillus plantarum*	TEM of EV isolates, protein characterization (MS) (Li et al., 2017)
	*Lactobacillus rhamnosus*	TEM of EV isolates (Behzadi et al., 2017)
	*Lactobacillus reuteri*	SEM and TEM of budding events or EV isolates (Grande et al., 2017)
	*Lactobacillus casei*	TEM and AFM of EV isolates, CLSM of budding events, protein characterization (SDS-PAGE, MS) (Domínguez Rubio et al., 2017)
	*Bifidobacterium longum*	TEM of EV isolates, protein characterization (SDS-PAGE, MS) (Kim J.H. et al., 2016)
	*Enterococcus faecalis*	TEM of EV isolates, protein characterization (SDS-PAGE, MS) (Kim J.H. et al., 2016)
Actinobacteria	*Mycobacterium tuberculosis*	TEM of budding events and EV isolates, protein characterization (MS), lipid characterization immunofluorescence (Prados-Rosales et al., 2014; Athman et al., 2015, 2017; Lee et al., 2015)
	*Mycobacterium smegmatis*	TEM of EV isolates, protein characterization (MS) (Prados-Rosales et al., 2011)
	*Mycobacterium avium*	TEM of EV isolates (Prados-Rosales et al., 2011)
	*Mycobacterium kansasii*	TEM of EV isolates (Prados-Rosales et al., 2011)
	*Mycobacterium phlei*	TEM of EV isolates (Prados-Rosales et al., 2011)
	*Mycobacterium bovis* Bacillus Calmette–Guérin (BCG)	TEM of budding events and EV isolates, protein characterization (MS), lipid characterization (Prados-Rosales et al., 2011)
	*Mycobacterium ulcerans*	SEM, protein characterization (MS) (Marsollier et al., 2007)
	*Streptomyces lividans*	TEM of culture supernatant, lipid staining, protein characterization (MS) (Schrempf and Merling, 2015)
	*Streptomyces coelicolor*	TEM of culture supernatant and EV isolates, cryo-EM and cryo-electron tomography, protein characterization (SDS-PAGE, MS) (Schrempf et al., 2011)


*TEM, transmission electron microscopy; SDS-PAGE, sodium dodecyl sulfate-polyacrylamide gel electrophoresis; MS, mass spectrometry; SEM, scanning electron microscopy; AFM, atomic force microscope; CLSM, confocal laser scanning microscopy.*

The expanding research field on Gram-positive EVs has so far revealed possible roles of EVs in bacterial ecology, physiology, and host–microbe interactions linked to health and disease depending on the bacterial species. In this light, EVs are also of potential value in medical and clinical applications. In this article we will provide new insights into the diversity, functionality, and possible applications of Gram-positive EVs.

## Physiological Roles of Gram-Positive EVs

The biogenesis mechanism of Gram-positive EVs was not instantly evident as for the outer membrane vesicles (OMVs) produced by Gram-negative bacteria. While OMVs are generated by pinching off the outer membrane, the generation and release of Gram-positive EVs through the thick cell wall is still being disputed. The current evidence-supported hypothesis involves the action of cell wall-degrading enzymes that weaken the peptidoglycan layer and facilitate the release of EVs (Brown et al., 2015; Toyofuku et al., 2017; Wang et al., 2018).

**FIGURE 1:**
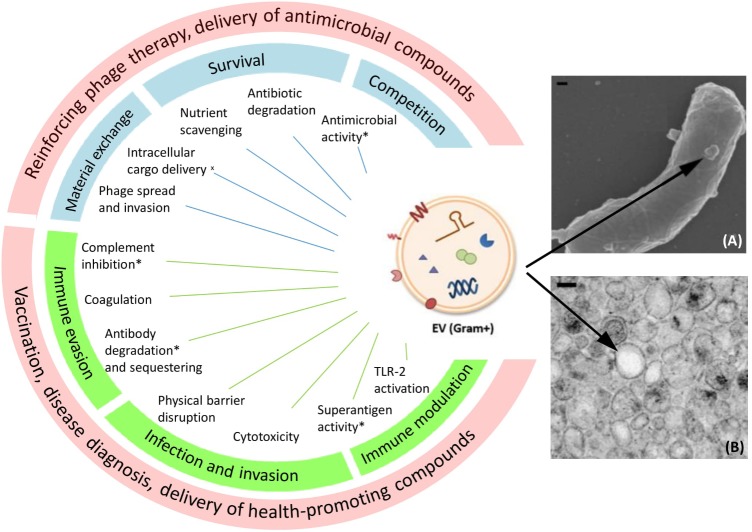
Proposed functions and potential medical applications of Gram-positive EVs. On the left side a schematic drawing of EVs carrying different types of cargo is shown. Functions marked with an asterisk (^∗^) have been proposed on the basis of EV protein content, but a functional effect remains to confirmed. (^x^) Bacterial cell–cell communication supported by membrane fusion assays. Blue boxes show EV roles in bacterial physiology and ecology, green boxes show EV roles in microbe–host interaction, and red boxes show proposed applications of EVs. On the right side a SEM picture **(A)** of EV production from *Bacillus subtilis* and a TEM picture **(B)** of isolated *B. subtilis* EVs are shown as examples (Brown et al., 2014). Copyright 2018 Wiley-Blackwell. EM pictures used with permission. Scale bars: 100 nm.

Similar to OMVs, Gram-positive EVs carry a wide range of cargo molecules including nucleic acids, proteins, lipids, viruses, enzymes, and toxins (Brown et al., 2015; Kim et al., 2015). Nevertheless, Gram-positive EVs can still be distinguished from OMVs since the latter typically contain lipopolysaccharide (LPS) and encapsulate periplasmic components. Experimental evidence has indicated that EV release is overall an active metabolic process and that dedicated sorting mechanisms are conceivably involved in determining the content of EVs (Prados-Rosales et al., 2011; Brown et al., 2014; Liao et al., 2014; Athman et al., 2015; Resch et al., 2016). This implies physiological or ecological importance of EV release in bacteria. An extensive overview of the possible physiological roles of Gram-positive EVs is presented in **Figure 1**.

Obviously, the type of cargo determines the role of EVs to a large extent. Gram-positive EVs have been suggested, among others, to play a role in horizontal gene transfer. In addition to the transfer of bacterial chromosomal DNA, which was revealed in *Ruminococcus* spp. (Klieve et al., 2005), EVs may also facilitate gene transfer by mediating bacteriophage (phage) infection. It was observed in *Bacillus subtilis* that phage-resistant cells acquired phage sensitivity by receiving phage receptors carried by EVs generated from susceptible bacteria (Tzipilevich et al., 2017). The same mechanism even enabled *B. subtilis* phages to attach to non-host species *Bacillus cereus* and *Bacillus amyloliquefaciens*, providing the initial step for phages to adapt to new hosts and exchange genetic material. Moreover, phage particles were found inside EVs (Toyofuku et al., 2017), and these EVs could potentially provide a novel route for phages to enter bacterial hosts, that is, their intracellular release following membrane fusion which was demonstrated in *B. subtilis* (Kim Y. et al., 2016). Thus, EV-mediated horizontal gene transfer among different bacterial strains and species, contributes to bacterial DNA transfer and to phage spreading and invasion (**Figure 1**).

Extracellular vesicles can also contribute to microbial survival or competition. To explicate the former, the scavenging properties of EVs support the uptake of nutrient molecules from the environment. For example, EVs derived from *Mycobacterium tuberculosis*, *Streptomyces coelicolor* as well as *Staphylococcus aureus* were shown to contain iron-binding factors that contribute to bacterial survival under iron-limited conditions (Lee et al., 2009, 2015; Schrempf et al., 2011; Jeon et al., 2016; Rodriguez and Prados-Rosales, 2016). Proteomic analysis also revealed the presence of beta-lactamase in *S. aureus* EVs. As a result, EVs produced by resistant bacteria could protect susceptible bacteria by degrading ampicillin in the environment (Lee J. et al., 2013). Although so far EV-conferred protection was restricted to this particular resistance factor, it is conceivable that EVs could be involved in the establishment of antibiotic resistant subpopulations via horizontal gene transfer and/or the transfer of antimicrobial factors. Competition is also believed to occur via antimicrobial factors released in EVs. The presence of autolysins in EVs points to a role in lytic attack on targeted bacteria (Olaya-Abril et al., 2014; Haas and Grenier, 2015; Lee et al., 2015) (**Figure 1**).

## Gram-Positive EVs in Bacteria–Host Interactions

In addition to the physiological roles of Gram-positive EVs, their functions in interacting with the human host are also revealed. Interestingly, Gram-positive EVs were shown to be internalized by eukaryotic cells via endocytosis in multiple epithelial and macrophage cell lines (Kim et al., 2012; Brown et al., 2014; Hong et al., 2014; Mulcahy et al., 2014; Surve et al., 2016). Moreover, the fusion of bacterial EVs with eukaryotic cell membranes has also been clearly demonstrated (Thay et al., 2013). In addition, EVs can directly interact with receptors on the surface of host cells to initiate intracellular signaling cascades (Kim et al., 2012; Prados-Rosales et al., 2014). Surface-exposed enzymes linked to EVs can function similarly to extracellular and cell surface-associated enzymes. Notably, it has been proposed that rupture of EVs facilitates targeted or delayed release of enzymes contained within vesicles, leading to a locally high and hence biologically active concentration of the released agent (Thay et al., 2013).

### EV-Associated Virulence

Since virulence factors can form a large constituent of the protein content of EVs, a strong interest is focused on the role of Gram-positive EVs during infection (Lee J. et al., 2013) (**Figure 1**). This not only relates to abundance, ranking among the top protein hits, but also the diversity of virulence factors found in EVs (Lee et al., 2009; Haas and Grenier, 2015) including so-called superantigens, capable of a-specifically activating a substantial portion of the human T cell repertoire (Lee J.H. et al., 2013; Jeon et al., 2016). Virulence factors specifically aimed at promoting invasion and spread throughout tissues have also been identified in EVs. Examples include collagenase and hyaluronate lyase that disrupts the extracellular matrix (ECM), and serine proteases, such as exfoliative toxins, which aid in the disruption of physical barriers (Jeon et al., 2016; Surve et al., 2016; Jeon et al., 2017). Functional effects of EV-incorporated virulence factors have so far most clearly been demonstrated for cytotoxic factors (Rivera et al., 2010; Thay et al., 2013). EVs produced by *Bacillus anthracis*, *S. aureus*, *Streptococcus pneumoniae*, *Streptococcus pyogenes*, and *Streptococcus agalactiae* were shown to carry a range of hemolysins and/or pore forming toxins (Rivera et al., 2010; Thay et al., 2013; Olaya-Abril et al., 2014; Jeon et al., 2016; Resch et al., 2016; Surve et al., 2016). Notably, the activity of such toxins can be altered or enhanced by enclosure inside or in membranes of EVs. Whereas soluble α-hemolysin induced apoptosis-like cell death, necrosis was caused following exposure to EV-enclosed α-hemolysin (Hong et al., 2014). This feature can be the result of a more preferable molecular organization of toxins in EVs or alternatively, by increased delivery to target cells.

Gram-positive EVs can contain an array of molecules involved in immune evasion (**Figure 1**). EVs of *S. aureus* bear coagulase enzymes and factors that can mediate clot formation upon addition of EVs to serum (Lee et al., 2009; Sugimoto et al., 2016). EVs can thus aid in the formation of fibrin networks surrounding pathogens, thereby forming a protective environment with limited access to the innate immune system. Also an efficient humoral immune response can be subverted by EVs. *M. tuberculosis*-derived EVs carrying lipoglycans were shown to inhibit T cell responses (Athman et al., 2017). Multiple protein-disrupting key steps of the complement cascade have been identified in EV preparations (Lee et al., 2009; Resch et al., 2016). In addition, an IgM protease as well as functional IgG binding factors could be retrieved from these samples (Lee et al., 2009; Gurung et al., 2011; Haas and Grenier, 2015). These factors would allow EVs to actively clear antibodies in the surroundings, in addition to their natural decoy ability due to antigenic similarity with the secreting pathogen.

### EVs in Clinical Disease

Given the virulence factors harbored in EVs, it is not surprising that EV exposure has been linked with the exacerbation or induction of a variety of disease states. For instance, the exposure of fetal–maternal structures to *S. agalactiae*-derived EVs can lead to fetal compromise and preterm termination of the pregnancy, as evidenced using mice models (Surve et al., 2016). Interestingly, EVs were able to travel along the female mouse reproductive tract toward the uterus. This phenomenon could provide an explanation for the paradoxical link between reproductive tract colonization and the occurrence of complications at the sterile fetal–maternal interface (Surve et al., 2016). These findings suggest that via the transfer of EVs, even infection or colonization at a distant site can contribute to disease development. Likewise, challenge with EVs can even occur via environmental exposure. *S. aureus* EVs were found in house dust, and in this form, bacterial products are thought to be more easily inhaled than whole bacteria (Kim et al., 2012). Incidental or repeated inhalation of *S. aureus* EVs was shown to cause airway inflammation in mice. Importantly, when EV exposure in the lungs was combined with allergens, a stronger sensitization occurred compared to the allergen exposure alone. EV exposure thereby enhanced a hypersensitivity response to the allergen in question (Kim et al., 2012). Gram-positive EVs therefore seem to represent an unforeseen contributor to frequently occurring, and seemingly unrelated disease conditions.

### EVs in Health Benefits

Although a number of studies focus on EVs derived from pathogens and hence associate them with health threats, evidence of EV production by probiotic bacteria is also emerging and drawing attention to the health benefits conferred by EVs (Ilinskaya et al., 2017; Liu et al., 2018). Strains of *Bifidobacterium longum*, *Lactobacillus rhamnosus*, *Lactobacillus casei*, and *Lactobacillus plantarum* were shown to produce EVs carrying effector molecules that are associated with the probiotic effects of the producing bacteria (Kim J.H. et al., 2016; Behzadi et al., 2017; Domínguez Rubio et al., 2017; Li et al., 2017). EVs from *B. longum* effectively alleviated food allergy response in a mouse model; purified *L. rhamnosus* EVs were shown to have significant cytotoxic effect on hepatic cancer cells; *L. casei*-derived EVs carry proteins that offer the host intestinal epithelial cells protective, anti-apoptotic effects; *L. plantarum*-derived EVs provided protection to the host against pathogenic bacteria. Often these effects could be observed with EVs but not with complete bacterial cells, possibly due to the fact that EVs can penetrate the intestinal epithelial barrier and migrate to the other organs or interact with the immune system of the host (Kim J.H. et al., 2016; Behzadi et al., 2017). These insights into the mechanisms of previously observed beneficial properties of probiotic microorganisms further highlight the importance of EVs.

## Repurposing Bacterial EVs in Medical Applications

Based on the revealed cargo components and speculated roles of Gram-positive EVs in microbe-microbe as well as microbe–host interactions, we propose several medical or biotechnological applications for Gram-positive EVs, including delivery of antimicrobial compounds, reinforcing phage therapy, vaccination, disease diagnosis, and delivery of health-promoting compounds (**Figure 1**).

### Delivery of Antimicrobial Compounds

The role of Gram-positive EVs in cargo exchange among bacteria suggests that they could serve to deliver compounds that have difficulties to pass the cell membrane, such as antibiotics. Liposome incorporation of the antibiotic tobramycin was shown to greatly increase the efficacy against *S. aureus*, implicating EV-mediated delivery of antimicrobials as an interesting therapeutic approach (Beaulac et al., 1998). Many strategies have been proposed for the artificial loading of vesicles, which could enable the delivery of antibiotics or other chemical compounds via natural EVs to enhance uptake and targeting compared to synthetic structures (Vader et al., 2016). Local concentration and simultaneous release of two or more compounds via EV-incorporation could enable synergy of drug actions. In the case of autolysins, joined release with other compounds might aid in the passage of drugs, or maybe even whole EVs, through the cell wall. As EVs offer protection to the cargos, they may indeed show advantage in delivery of compounds susceptible to degradation, for example antimicrobial peptides (Moncla et al., 2011).

### Reinforcing Phage Therapy

Phage therapy is being revisited frequently nowadays as an alternative to antibiotics for treating bacterial infections due to the increasing severity of antibiotic resistance (Nobrega et al., 2015; Lin et al., 2017). However, a challenge posed to phage therapy is the narrow host-range of phages (Koskella and Meaden, 2013). To tackle this, the use of phage cocktails (Sadekuzzaman et al., 2017; Yen et al., 2017) or engineering phages with broad host ranges has been reported (Ando et al., 2015; Pires et al., 2016). As Gram-positive EVs can have a role in promoting the spread of phages and invasion of novel bacterial hosts by transferring phage receptors (Tzipilevich et al., 2017), EVs might provide extra opportunities in phage therapy too. EVs derived from phage-sensitive bacteria can be administered prior to the phages, to enhance the targeting of bacteria and even enable the infection of novel bacterial host targets.

Another challenge in phage therapy is the reduction in activity in the case of orally administered phages through the gastrointestinal tract (GIT), where factors such as low pH have a large impact on phage activity (Ly-Chatain, 2014). Liposome encapsulation was shown to improve phage stability in GIT both in simulated gastric fluid and *in vivo* (Colom et al., 2015). Similarly, we speculate that EV-engulfed phages may demonstrate improved tolerance to hostile conditions, and therefore show higher efficacy after (oral) administration. In addition, EV-mediated transfer of phages themselves could serve as a potential receptor-independent delivery route, although this theory remains to be confirmed experimentally.

### Vaccination

Since EVs can transport bacterial products to the host, large efforts are being undertaken to study the EV-mediated delivery of immunogenic antigens. Initial steps toward the clinical use of Gram-positive EVs as disease prophylaxis are currently being evaluated. A patent has already been filed claiming ownership of the method of immune induction via Gram-positive EVs, thereby aiming to develop a new vaccination strategy (Gho et al., 2016). So far, beneficial effects of vaccination with Gram-positive EVs have been demonstrated for *Clostridium perfringens*, *S. pneumoniae*, *B. anthracis*, *M. tuberculosis*, and *S. aureus*. In mice models, the above mentioned EV vaccination prolonged survival time or increased the survival rate upon lethal challenge (Rivera et al., 2010; Jiang et al., 2014; Olaya-Abril et al., 2014; Choi et al., 2015). In addition, the bacterial burden to the host immune system and the degree of inflammation could be reduced upon a sub-lethal challenge (Prados-Rosales et al., 2011; Choi et al., 2015). While, Wang et al. (2018) employed genetic engineering to ensure the production of non-toxic *S. aureus* EVs as effective vaccines in mice, another study showed that also non-engineered *S. aureus* EVs did not cause notable toxicity in mice despite the potent immune activation (Choi et al., 2015). This is in contrast to OMV-based vaccines, for which toxicity of LPS constituents hampers the vaccine application (Acevedo et al., 2014). Gram-positive EVs might thus form an even more potent vaccination strategy than currently licensed vaccines.

### Disease Diagnosis

The link between gut microbiota composition and human health or diseases is being revealed gradually in recent years (Marchesi et al., 2016; Yamashiro, 2017; Chelakkot et al., 2018). Studies show that EVs derived from the gut microbiota are distributed throughout the human body including the blood and urine, and they reflect the composition of the microbiota to a great extent (Kang et al., 2013; Jang et al., 2015; Yoo et al., 2016). This observation opens doors to novel methods of disease diagnosis or assessment. As an example, Lee et al. (2017) successfully developed a rapid, non-invasive assessment method on microbiota profiles in autism spectrum disorder patients by examining the 16S rRNA gene sequences in bacterial EVs isolated from urine samples. Since EV release is often the result of active metabolism in bacteria, EVs may form a better indication of the microbiota activities in the hosts than the bacterial populations themselves, and therefore provide more insights into the links between microbiota and the disease or health status of the hosts.

### Delivery Vehicles of Health-Promoting Compounds

As EVs were shown to play important roles in realizing the anti-allergy, anti-inflammation, and cancer-inhibiting effects of several probiotic bacteria (Kim J.H. et al., 2016; Behzadi et al., 2017; Li et al., 2017), the opportunity of using EVs as a booster or even substitute for bacteria to achieve probiotic effects becomes attractive. Moreover, EVs may offer protection and serve to deliver beneficial nutritional compounds, namely proteins or vitamins, to the hosts in an efficient manner (Liu et al., 2018). The same effect might not be attained by ingesting the pure compounds or whole-cell bacteria due to degradation of the effector molecules or limited accessibility to the targeted tissue or cells. Therefore, bacterial EVs may contribute to novel formulations of probiotics or food supplements.

## Concluding Remarks

Gram-positive bacteria constitute a large and widely diverse group, including species that are extensively used in food fermentations and as probiotics, whereas other species are known to be pathogenic causing a range of foodborne and clinical infections. So far, a clear picture has emerged showing that Gram-positive EVs may play a role in a wide range of biological events and consequently in human health and disease. These initial indications form a strong framework that can guide new research lines focused on the mechanistic understanding of these events and the translation of individual examples to general concepts and applications. Yet, many enigmatic puzzles regarding the underlying biology of Gram-positive EVs still remain unsolved, especially in EV biogenesis and uptake. Elucidation of these aspects may further stimulate innovative medical and biotechnological applications.

## Author Contributions

YL and KD wrote the manuscript. ES and TA critically reviewed the manuscript and provided feedback. All authors have read and approved the final version of the manuscript.

## Conflict of Interest Statement

The authors declare that the research was conducted in the absence of any commercial or financial relationships that could be construed as a potential conflict of interest.
